# Broad application prospects of bone turnover markers in pediatrics

**DOI:** 10.1002/jcla.24656

**Published:** 2022-08-10

**Authors:** Yiduo Zhang, Xiaocui Huang, Chao Li, Jing Zhang, Xingnan Yu, Ye Li, Wenjie Zhou, Fan Yu

**Affiliations:** ^1^ Department of Laboratory Medicine West China Second University Hospital, Sichuan University Chengdu China; ^2^ Key Laboratory of Birth Defects and Related Diseases of Women and Children (Sichuan University) Ministry of Education Chengdu China; ^3^ Chengdu Jinjiang District Maternal and Child Healthcare Hospital Chengdu China

**Keywords:** ALP, bone turnover markers, clinical utility, CTX, metabolic disease, osteocalcin, pediatrics, PINP

## Abstract

**Background:**

Bone turnover markers (BTMs) have been studied for application in clinical medicine. However, BTMs in children are challenging, and few studies explore these BTMs in children. The application of BTMs is complicated mainly due to pre‐analytical factors, variable reference intervals of age‐ and sex‐related BTMs for adolescents and children in different regions and laboratories. Therefore, laboratory testing of BTMs is critical for understanding pediatric bone development and metabolism, which provides additional information about bone development and diseases.

**Methods:**

Literature search was conducted using the MeSH term “child” combined with the terms that bone turnover markers such as “osteocalcin,” “Procollagen type I N‐terminal propeptide,” “procollagen type I C‐terminal propeptide,” “osteocalcin,” “N‐terminal cross‐linked telopeptide,” and “C‐terminal cross‐linked telopeptide,” Several databases including Web of Science, Google Scholar, and PubMed were searched to obtain the relevant studies.

**Results:**

BTMs represent the combined effects of skeletal development, growth, and remodeling in children, which can be used in clinical pediatrics to assist in the diagnosis and prognosis of bone metabolic disorders.

**Conclusion:**

BTMs are clearly helpful for diagnosis and monitoring of bone growth and development as well as bone metabolic disorders.

## Graphical abstract

Bone turnover markers (BTMs) have been evaluated for use in medicine; however, there are challenges to the interpretation of BTMs in children, in part, because there have been too few studies in children. BTMs represent the combined effects of the formation, growth, and remodeling of bone metabolism in children. The use of BTMs is complicated primarily due to pre‐analytical factors and variable reference intervals of age‐ and sex‐related BTMs for adolescents and children in different regions and laboratories. BTMs may be used for diagnosis and prognostic prediction of bone metabolic disease to support clinical pediatrics. Laboratory testing of BTMs is important for understanding pediatric bone development and metabolism, which provides additional information about bone development and disease. BTMs are useful for the diagnosis and monitoring of bone growth and development and bone metabolic disorders.

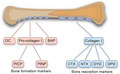

## 1 INTRODUCTION

Osteoporosis is a prevalent condition in the middle‐aged and elderly population, due to insufficient bone mass during childhood and adolescence.^1^ The level of peak bone mass acquired in adolescence and the rate of bone loss in adulthood are two important factors of osteoporosis. Peak bone mass attained in adolescence determine the likelihood of pathological fractures and osteoporosis in adulthood.^2^ People who do not attain adequate bone mass during childhood and adolescence are at risk for osteoporosis, even if they do not have accelerated bone loss in adulthood. Thus, even if osteoporosis does not occur in childhood, the achievement of bone mass during adolescent development can influence skeletal development in adulthood.

Dual‐energy X‐ray absorptiometry (DXA) and quantitative computed tomography (CT) using densitometry techniques are recognized as gold standards for assessing bone mineral contents and density for children. Normally, DXA and QCT had low radiation exposure, with the advantages of high precision and fast detection. However, there are some problems when using DXA and QCT to diagnosis for children, such as fussiness, irritability, and crying during their examination, resulting in poor reproducibility, and inability to reflect the real‐time biology of the bone at that time point.^3^, ^4^ Therefore, BMD measurements have limited the use of skeletal health assessment in childhood growth disorders for a long time.

As they are minimally invasive and can be dynamically monitored, bone metabolic marker assays have been widely used in the field of childhood growth and development‐related diseases, and their levels not only reflect the skeletal metabolic health of children but indirectly reflect the growth and development of children. Thus, BTMs provide a basis for early identification, diagnosis, and treatment monitoring of childhood growth and development diseases.

In this article, the characteristics of BTMs are discussed, including their classification, application in monitoring bone growth in children and adolescents. This review also discusses the relevant important studies and application of bone metabolism biomarkers in energy metabolism, the endocrine environment, osteoporosis, and childhood diseases.

## 2 BONE TURNOVER MARKERS

### 2.1 Specific BTMs provided by clinical laboratories

**FIGURE 1:**
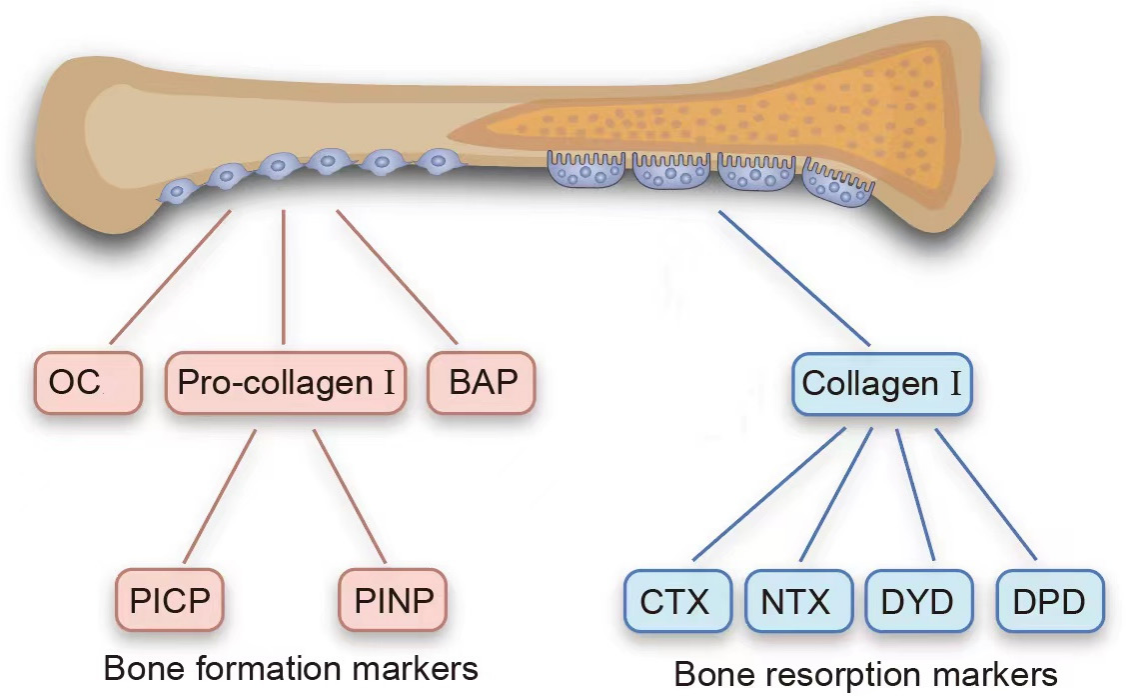
Biochemical markers of bone remodeling. BAP, bone alkaline phosphatase; CTX, C‐terminal cross‐linked telopeptide; DPD, deoxypyridinoline; NTX, N‐terminal cross‐linked telopeptide; OC, osteocalcin; PICP, procollagen type I C‐terminal propeptide; PINP, procollagen type I N‐terminal propeptide; PYD, pyridinoline.

Bone turnover markers **(**BTMs) are biochemical or cellular compounds produced during the continuum of bone resorption or formation.^5^ BTMs can be divided into two categories: bone formation and bone resorption markers (Figure 1 and Table 1). The former represents the activity of osteoblasts and the state of bone formation, while the latter mainly reflects the activity of osteoclasts and the level of bone resorption. Procollagen type I N‐terminal propeptide (PINP), procollagen type I C‐terminal propeptide (PICP), and osteocalcin (OC) are three frequently used markers of bone formation. Pyridinoline (PYD), deoxypyridinoline (DPD), N‐terminal cross‐linked telopeptide (NTX), and C‐terminal cross‐linked telopeptide (CTX) are bone resorption markers. In addition, some important cytokines implicated in the regulation of bone turnover by controlling the activity of osteoblasts or osteoclasts, such as osteoprotegerin (OPG) and receptor activator of nuclear factor κB ligand (RANKL), and these can be considered regulators of bone turnover rather than classical biochemical markers of bone turnover.

**TABLE 1:** Commonly measured serum and urinary biochemical bone resorption or formation markers

	Marker	Acronyms	Clinical source	Assay	Kidney‐cleared
Bone formation	Procollagen type I N‐terminal propeptide	PINP	Serum	ELISA, ECLIA	No
	Procollagen type I C‐terminal propeptide	PICP	Serum	IRMA, ELISA	No
	Osteocalcin	OC	Serum	ELISA, ECLIA	Yes
	Bone alkaline phosphatase	BAP	Serum	IRMA, ELISA	No
bone resorption	Pyridinoline	PYD	Urine or serum	HPLC, ELISA	Yes
	Deoxypyridinoline	DPD	Urine or serum	HPLC, ELISA	Yes
	N‐terminal cross‐linked telopeptide	NTX	Serum or urine	ELISA, ECLIA	Yes
	C‐terminal cross‐linked telopeptide	CTX	Serum or urine	ELISA, ECLIA	Yes

Abbreviations: ECLIA, electrochemiluminescence immunoassay; ELISA, enzyme‐linked immunosorbent assay; HPLC, high‐performance liquid chromatography; IRMA, immunoradiometric assay.

As BTMs are mostly excreted by the kidneys, their levels are frequently detected in urine.^6^ Due to the difficulty of collecting 24‐hour urine samples and the need to adjust for creatinine, blood testing is a preferable sample collection technique over urine sampling.^5^ Laboratory testing of BTMs is minimally invasive and relatively affordable and, if used and interpreted properly, it can be a useful tool for assessing metabolic bone disease, treatment outcomes, and patient compliance.^7^, ^8^ CTX and PINP are the commonly used bone turnover markers in clinical research and are recommended by the International Osteoporosis Foundation (IOF) and the International Federation of clinical laboratory medicine (IFCC).^9^ With the development of fully automated platforms, the analytical variability of bone markers has been greatly improved. The European Federation of Clinical Chemistry and Laboratory Medicine (EFLM) has shown the biological variability (CV%) of some BTMs, such as ALP, PINP, CTX, and osteocalcin, and with small interlaboratory variation.^10^ In this review, we describe several of the most widely used BTMs in clinical pediatrics.

### 2.2 Procollagen type I N‐terminal propeptide (PINP)

Type I collagen is a procollagen secreted by osteoblasts, which creates a triple helix consisting of PINP and PICP. Since these propeptides are cleaved in their extracellular area and released into the circulation as metabolites, PINP and PICP concentrations can represent the level of bone formation.^11^ Although the clearance of PINP may be less susceptible to hormonal changes than PICP,^12^ PINP is a more sensitive marker compared with PICP.^13^ Several studies have noted that PINP remains stable even after repeated freezing and thawing,^6^ and its levels are not affected by circadian rhythms, so it is not necessary to consider the timing of sampling.^14^

### 2.3 Osteocalcin

Osteocalcin is the most common noncollagenous protein in bone, and it is secreted primarily by mature osteoblasts, although it is also secreted during bone resorption. Thus, serum osteocalcin is an important indicator of bone turnover, indicating bone formation and bone resorption.^6^, ^15^ Because serum osteocalcin concentrations have a distinct circadian rhythm and are highest in the morning,^16^ blood samples must be taken in the morning to ensure the accuracy and comparability of the test results. In addition, several studies have shown that seasonal changes and diet did not affect PINP or osteocalcin levels.^17^, ^18^, ^19^, ^20^

### 2.4 Bone alkaline phosphatase (BALP)

Human ALP is classified as tissue non‐specific ALP (TNSALP), intestinal type, placental type, and placental‐like type.^21^ TNSALP activity is represented by the products of the ALPL gene, which indicates bone anabolic activity. ALPL gene mutation leads to abnormal skeletal mineralization. BALP is a common biochemical marker of bone formation and a specific marker for osteogenesis, as well as one type of TNSALP, along with liver‐ and kidney‐type ALP. The expression of bone ALP occurs early in the development of osteoblasts from mesenchymal progenitors and is vital in the degradation of pyrophosphate, a natural inhibitor of mineralization.^15^ It is hard to identify bone‐type from liver‐type ALP using current immunoassays, as they share the same amino acid sequences. Clinically, the application and interpretation of bone ALP as a BTM should be unaffected by liver diseases because bone ALP is normally cleared from the serum by liver.^22^ Children have higher bone ALP activity than adults due to higher bone formation rates. Determination of bone ALP activity also seems to be helpful for the diagnosis of hypophosphatasia and hyperphosphatasia.^23^

### 2.5 C‐ and N‐Terminal Telopeptides of Type I Collagen (CTX and NTX)

During bone degradation, osteocalcin breaks down the bone matrix and releases CTX and NTX.^8^ Although both CTX and NTX can be measured from urine samples, CTX has gained prominence because it can also be determined from blood tests on some automated platforms. Additionally, it is the preferred biomarker for detecting bone resorption activity.^6^ The designed method determined the particular amino acid sequence of telopeptide type I collagen is known as cross lap, and β‐aspartic acid was called β‐CTX.^18^ The International Osteoporosis Foundation recommends CTX as an appropriate bone marker for investigating bone resorption in clinical and research settings.^24^ Since circadian rhythms and food consumption have effects on circulation β‐CTX,^25^ it should be collected during the period of fasting in the morning.^17^

## 3 APPLICATION OF BTMs

Many clinical and osseous manifestations of metabolic bone disease are more prevalent in children. However, too few studies on the use of these BTMs in children have potential use implications. Here, we summarize the role of BTMs in clinical pediatric care.

### 3.1 Application of BTMs in growth and development

Children and adolescents are important periods of skeletal growth and exhibit high rates of bone growth and rapid bone turnover. BTMs reach the first peak within 1 year after birth, with little differences between boys and girls. Then, BTMs begin to show a downward trend and reach the second peak in early adolescence at the age of eight, with gender differences.^26^ The peak of BTMs occurring in girls is earlier than that in boys, while the magnitude of the peak was lower than in boys. This could be related to secondary sexual characteristics and hormone levels. The decline in BTMs levels occurred earlier in girls than in boys and was more significant than in boys during late adolescence, which can explain the differences in bone peak and bone mineral content (PBC) between boys and girls during puberty.^27^ A comparison of the reference interval ranges of BTMs in adults and children showed that the levels of BTMs were higher in childhood than those in adults and did not approach adult levels until late adolescence.^26^, ^28^, ^29^, ^30^, ^31^, ^32^

Many factors can influence childhood growth and development, including genetics, nutrition, endocrine status, medication usage, and tumors. These disorders directly or indirectly affect PBC in childhood. Several studies have evaluated the relationship between BTMs and skeletal growth,^33^, ^34^, ^35^, ^36^ and BTMs may be a strong predictor of skeletal status in childhood and adolescence, as well as a good predictor of future skeletal growth. Serum BTM levels in CDGP boys were found to be comparable to those of healthy children.^34^, ^36^ Meanwhile, PINP, OC, and CTX values are lower in preschool and school age, decline during adolescence, and decrease rapidly after puberty, similar to the growth characteristics of children.^26^, ^28^, ^29^, ^30^, ^31^, ^32^ Gascoin et al.^37^ found that idiopathic short stature (ISS) children had lower PINP concentrations than normal children of the same age, and that height growth correlated with PINP concentrations during the first year of GH treatment. In addition, a substantial positive correlation among BAP, OC, and insulin‐like growth factor 1 (IGF‐1) in children with ISS.^37^ BAP and OC reflect skeletal growth dynamics and skeletal growth outcomes and can be used as monitoring indicators to assess the current growth status of children with ISS and to monitor treatment effects.

### 3.2 Application of BTMs in rickets

Rickets is a childhood disorder associated with mineralization and ossification defects, the most common of which is vitamin D deficiency.^38^ Despite significant improvements in early screening and quality of life, epidemiological surveys have revealed that the prevalence of nutritional rickets in children in rural areas remains as high as 10%.^39^, ^40^ Due to the dramatic increase in serum ALP in children with this disease, studies have confirmed the use of total serum ALP or BALP as an early screening indicator for differential nutritional rickets due to the good correlation between total serum ALP and BALP in childhood, with normal levels suggesting a low likelihood of rickets.^39^, ^41^ In addition, Chatterjee et al.^42^, ^43^ discovered that ALP is strongly expressed in nutritional rickets and is a more reliable marker than osteocalcin, PICP, and NTX and that its expression level may be utilized clinically to predict disease severity and prognosis.

However, P1NP, β‐CTX, PTH, and 25(OH)D3 have hardly been studied in the field of nutritional rickets in children, and their application value needs to be further discovered and evaluated.

### 3.3 Primary osteoporosis

Children's primary osteoporosis is a genetic disease caused by mutations, and osteogenesis imperfect is the most common disease caused by 17 identified genetic defects.^44^ The main manifestation is increased bone fragility.^45^, ^46^ Moreover, Abdulmoein^47^ showed reduced CTX and osteocalcin levels in children with primary osteoporosis 3 months after the treatment with zoledronic acid, suggesting that bone metabolism can be inhibited via bone resorption and short‐term side effects after early treatment. However, due to their limited predictive value for the diagnosis of fractures, BTMs are not currently used in the diagnosis of osteoporosis. It would be possible to monitor the rate of bone loss throughout drug therapy if baseline BTM levels were compared with follow‐up values.

### 3.4 Secondary osteoporosis

Secondary osteoporosis can be caused by multiple factors, such as primary disease and associated therapy. Harada and Rodan firstly found that osteoblasts and osteoclasts release active compounds are important to the physiological activity of other organs.^48^ Previous studies have indicated that bone is not only the structural scaffold of the human body, but also can be important endocrine and hormone target organ.^49^, ^50^ In addition, many young patients develop secondary osteoporosis due to chronic diseases and the medications used.^51^

#### 3.4.1 Application of BTMs in diabetes

According to the International Diabetes Federation, Type 1 diabetes (T1D) is most common in children and adolescents, with more than 130,000 people under the age of 20 diagnosed each year. Bone has been identified as an endocrine organ that regulates glucose and energy metabolism. According to research conducted by Schwartz,^52^ enhanced glycemic management reduced the incidence of fractures associated with osteoporosis, suggesting that OC is a highly sensitive biomarker of the bone conversion process, which is reduced in patients with poor glycemia. This may be related to the fact that OC regulates energy metabolism. Thus, osteocalcin may reflect early alterations in bone metabolism in diabetic patients and may serve as an indicator of bone turnover.

#### 3.4.2 Application of BTMs for obesity

Childhood obesity is a chronic nutritional disease caused by excessive body fat accumulation. According to the World Health Organization (WHO), the number of overweight or obese children under the age of five reached 60 million worldwide in 2020, and the number of obese children aged 0–7 years in China reached 5.31 million, with the trend continuing to rise.^53^ Although studies have shown that the incidence of osteoporosis and fracture risk is significantly higher in children with obesity compared with healthy children, it is worth noting that DXA found no differences in osteoporosis between the two,^54^ implying that monitoring bone metabolic status is critical for the bone health of children with obesity. Obese children had significantly lower levels of calcium, phosphorus, ALP, 25(OH)D3, P1NP, and OC than healthy children of the same age, as well as lower mean height than normal children of the same age.^55^, ^56^ Obesity in children not only affects normal growth and development but also leads to metabolic diseases such as diabetes. A meta‐analysis confirmed that OC was reduced in almost all children with type 1 diabetes, and OC was negatively correlated with glycosylated hemoglobin.^57^, ^58^ The OC may be related to the regulation of glucose metabolism and bone metabolism, as it can reduce the incidence of osteoporosis‐related fractures by improving blood glucose. Although there are no follow‐up studies on the risk of osteoporosis in obese children and adolescents in early adulthood, numerous studies have confirmed the importance of BTMs for assessing bone health levels in children with obesity and metabolic diseases, as well as aiding in the diagnosis and treatment monitoring.

#### 3.4.3 Application of BTMs in hematological diseases and bone tumors hematologic diseases

Hematologic disorders (acute lymphocyte leukemia (ALL), thalassemia, and idiopathic thrombocytopenic purpura (ITP)) and bone‐tumor (multiple myeloma and osteosarcoma) have a direct impact on the skeleton of pediatric patients.^59^, ^60^, ^61^, ^62^, ^63^ Bone has long been recognized as the most common target organ for malignant metastases, and skeletal cell metastases can lead to osteolysis. DXA measurements of bone mass would be underestimated due to the lack of growth hormone or a delay in pubertal development. Young adults suffer a considerable loss in BMD and insufficient density of bone minerals, which may be related to cancer itself, its therapy, or complications such as endocrine problems (decreased gonadal function, growth hormone shortage, etc.), all of which contribute to reduced bone mass gain. Some ALL children have significant osteoporosis at the time of diagnosis, and most of them will acquire this condition during the therapy process. Osteoporosis might occur during the diagnosis in some therapy cases of ALL children, and the majority will acquire this condition during the therapy process. Within 24 months of chemotherapy maintenance, 64% of children had a reduction in bone mineral content (BMC), and children showed alterations in bone conversion with osteocalcin and CTX.^64^, ^65^ In addition, young adult survivors of ALL had a decrease in BMD because of the possibility of the direct impacts of chemotherapy, steroids, or both on the skeleton during childhood and thus on the increase in bone mass.^66^ In the long run, Delvin's view is that 251 individuals who had been cured of leukemia did not show abnormalities in bone turnover markers.^67^ For thalassemia major patients, the decrease in osteocalcin may be due to osteoblastosis caused by iron overload. Nevertheless, no significant difference was found in serum alkaline phosphatase levels.^68^ In another research group, normal 25(OH)D concentrations may maintain normal calcium homeostasis in patients with thalassemia, suggesting that a normal vitamin D level is important in the pathogenesis of thalassemia bone disease.^69^ Furthermore, in patients with chronic ITP, OC, and type I collagen C‐terminal propeptide (PICP) concentrations were lower, urine DPD output was higher, and bone mineral density (BMD) was significantly lower in both the spine and hip Z‐scores.^70^ Moreover, BALP is statistically significantly increased with osteosarcoma in patients, while N‐MID osteocalcin and CTX are not significantly different in adolescent and adult groups. Nonetheless, high BALP has the diagnosis value of adult osteosarcoma but is not recommended for the differential diagnosis of adolescent patients, as BALP is affected by age, pubertal stage, and growth rate. Both N‐MID osteocalcin and CTX have limited use in the differential diagnosis of primary bone tumors.^71^ However, since there have been so few reports of solid tumors in children, it has to be studied further.

#### 3.4.4 Application of BTMs in Juvenile Rheumatoid Arthritis (JRA)

Juvenile rheumatoid arthritis (JRA) is typically connected with osteoporosis, particularly in children's long bones. In addition to impaired skeletal health, children with rheumatic disorders have other risk factors such as delayed growth, malnutrition, decreased weight‐bearing exercise, inflammation, and glucocorticoid medication.^72^ Children with RA almost generally fail to produce adequate bone mineralization, as indicated by bone formation failure and lower ICTP.^73^, ^74^ Inflammation is a crucial factor in the development of osteoporosis, and the majority of research on the OPG/RANKL/RANK axis in rheumatic disorders has been conducted on adults with rheumatoid arthritis (RA). Several studies have found that the degree of osteoporosis in children with chronic arthritis can be affected by the intensity of inflammation, even in the absence of corticosteroid treatment.^72^ Meanwhile, high RANKL concentrations are seen in acute RA.^75^ Osteoblasts and osteocytes secrete RANKL, which is also produced by B lymphocytes. It facilitates bone resorption and plays an important role in osteoclastogenesis.^76^ There has been a reduction in bone formation markers including osteocalcin and bone ALP.^73^

## 4 PROSPECTS

BTMs are important indicators to evaluate bone health status, and their assays are minimally invasive and reproducible. They are currently utilized in clinical practice to diagnose osteoporosis, predict fracture risk, evaluate treatment effects, determine tumor bone metastases, etc. The role of the bone as an endocrine organ is also being recognized by medical researchers due to the close connection between bone metabolism and glucose metabolism. Furthermore, BTMs played important role in the research field of childhood growth and development‐related diseases. Moreover, BTMs are being used for early screening, diagnosis, and monitoring of the efficacy of growth and development‐related diseases for children. However, the relationship between BTMs and DXA, the gold standard for clinical measurement of bone mineral density in children, is still limited to correlational studies, and the clinical use of their combined testing remains to be investigated further. Therefore, we need to further study to make sure a reliable combination of them in clinical application. We look forward to conducting more analysis on the detection and application of BTMs in children and adolescents, this will enable us to have a more comprehensive application in the growth and prevention of bone metabolic disorders. Therefore, pediatrician should be pay more attention on the use of BTMs in pediatrics.

## FUNDING INFORMATION

This work was supported by the Sichuan Province Science and Technology Support Program (Grant No. 2020YFS0107) and the Chengdu Science and Technology Support Program (Grant No. 2021‐YF05‐01500‐SN).

## CONFLICT OF INTEREST

The authors declare that they have no competing interests.

## Data Availability

Data sharing not applicable to this article as no datasets were generated or analysed during the current study
